# Experimental neoichnology of post-autotomy arm movements of sea lilies and possible evidence of thrashing behaviour in Triassic holocrinids

**DOI:** 10.1038/s41598-020-72116-1

**Published:** 2020-09-15

**Authors:** Przemysław Gorzelak, Mariusz A. Salamon, Krzysztof Brom, Tatsuo Oji, Kazumasa Oguri, Dorota Kołbuk, Marek Dec, Tomasz Brachaniec, Thomas Saucède

**Affiliations:** 1 Institute of Paleobiology, Polish Academy of Sciences, Warsaw, Poland; 2 Faculty of Natural Sciences, University of Silesia in Katowice, Sosnowiec, Poland; 3 University Museum, Nagoya University, Furo-cho, Nagoya, 464-8601 Japan; 4 Japan Agency for Marine-Earth Science and Technology (JAMSTEC), 2-15 Natsushima-cho, Yokosuka, 237-0061 Japan; 5 Polish Geological Institute – National Research Institute, Warsaw, Poland; 6 Biogéosciences UMR CNRS 6282, Université Bourgogne Franche-Comté, Dijon, France

**Keywords:** Palaeoecology, Palaeontology

## Abstract

Echinoderms exhibit remarkable powers of autotomy. For instance, crinoids can shed arm and stalk portions when attacked by predators. In some species, it has been reported that the autotomized arms display vigorous movements, which are thought to divert the attention of predators. This phenomenon, however, has not been well explored. Here we present results of experiments using the shallowest water species of living stalked crinoid (*Metacrinus rotundus*) collected at 140 m depth. A wide range of movements of detached arms, from sluggish writhing to violent flicks, was observed. Interestingly, autotomized arms produce distinct traces on the sediment surface. They are composed of straight or arched grooves usually arranged in radiating groups and shallow furrows. Similar traces were found associated with detached arms of the oldest (Early Triassic) stem-group isocrinid (*Holocrinus*). This finding may suggest that the origins of autotomy-related thrashing behaviour in crinoids could be traced back to at least the Early Triassic, underscoring the magnitude of anti-predatory traits that occurred during the Mesozoic Marine Revolution. A new ethological category, autotomichnia, is proposed for the traces produced by thrashing movements of shed appendages.

## Introduction

Many animals, including echinoderms, are able to autotomize parts of their body usually as a defense strategy against predators^1^. It has been argued that shed appendages, which sometimes display vigorous post-autotomy movements, reduce the animal’s mortality in two major ways: (1) they enable the animal to break away from predators that have grasped it, and (2) divert the attention of the predators away from the vulnerable body parts^2^.

Crinoids commonly referred to as sea lilies, a group of echinoderms that is subject to a high predation pressure, have remarkable ability to autotomize and regenerate their appendages^3–6^. It has been shown that arm autotomy in these echinoderms is achieved through the nervously mediated (L-Glutamate invoked) destabilisation of collagenous ligamentary fibres at specialized autotomy planes, namely (crypto)syzygial articulations^7^. Although considerable effort has been devoted to study autotomy in crinoids^8–11^, little attention has been paid to post-autotomy thrashing behaviour. Wilkie et al.^7^ in their study on living stalkless comatulids (feather stars) only briefly reported that: “after autotomy induced in both intact animals and isolated arms, the detached distal portion of the arm showed rhythmical cycles of flexion and extension in the oro–aboral plane”.

To further examine thrashing behaviour in extant sea lilies, and explore its ichnological potential we conducted neoichnological aquarium experiments using the stalked crinoid *Metacrinus rotundus* Carpenter, 1884. We then analyzed samples with articulated arm fragments of the oldest (Early Triassic) stem-group isocrinids (holocrinids) in order to identify evidence of this behaviour in the fossil record.

## Aquarium experiment

Specimens of living stalked crinoids of *Metacrinus rotundus* were used in this study. Sampling and handling of these specimens followed described procedure^12–14^. Crinoids were dredged from Suruga Bay (near the town of Numazu, Shizuoka Prefecture; Japan; ~ 35° 3′ N, ~ 138° 48′ E, ~ 140 m depth) from the sea bottom using a 90-cm-wide naturalist dredge with a net. Then, the living specimens were transferred to an experimental seawater tank in the Nagoya University Museum. The aquarium was maintained at a constant seawater temperature (~ 16 °C) in darkness and under circulation provided by a water pump. After few weeks of acclimatizing the crinoids, a box (~ 40 × 30 cm) floored with fine-grained sand was placed, which was smoothed before an autotomized arm was introduced. Autotomy of crinoid arm was induced following described procedure^15,16^. Neoichnological experiments were repeated several times using different arms from different individuals (electronic supplementary movies S1–S4).

For comparison purposes, a series of aquarium experiments was made on production of transport-induced sole markings left on the sediment surface (fine-grained sand) by isolated dead arms being dragged (with the pinnules facing upstream or downstream; electronic supplementary movies S5, S6) by a current (0.35 cm/s) adjusted by a water pump that was placed at the bottom of the aquarium. At the lower or higher velocity, the arms (which were placed at a distance of ~ 25 cm from the pump) were not moving or were lifted above the sediment surface, respectively, leaving no traces. These experiments were conducted at the Laboratory of Experimental Taphonomy at the Faculty of Natural Sciences of the University of Silesia in Katowice, Sosnowiec, Poland.

Gypsum casts from both experiments are deposited at the Institute of Paleobiology, Polish Academy of Sciences (ZPALV.42ICH).

## Fossil samples

A Lower Triassic (lowest Spathian) slab of thin-bedded silty limestone of the Thaynes Group (west of Paris, Idaho, USA) preserving five isolated fragments of arms belonging to one of the oldest (if not the oldest) post-Paleozoic holocrinid taxon (*Holocrinus* sp.) deposited in the collection of the Université de Bourgogne, Géologie Dijon, France (UBGD 30564) was investigated^17^. These arm fragments are of different lengths and lack distal arm tips and some distal pinnules (see figs. 2 and 3 in^17^).

## Results

### Traces of recent crinoid arms

**Figure 1:**
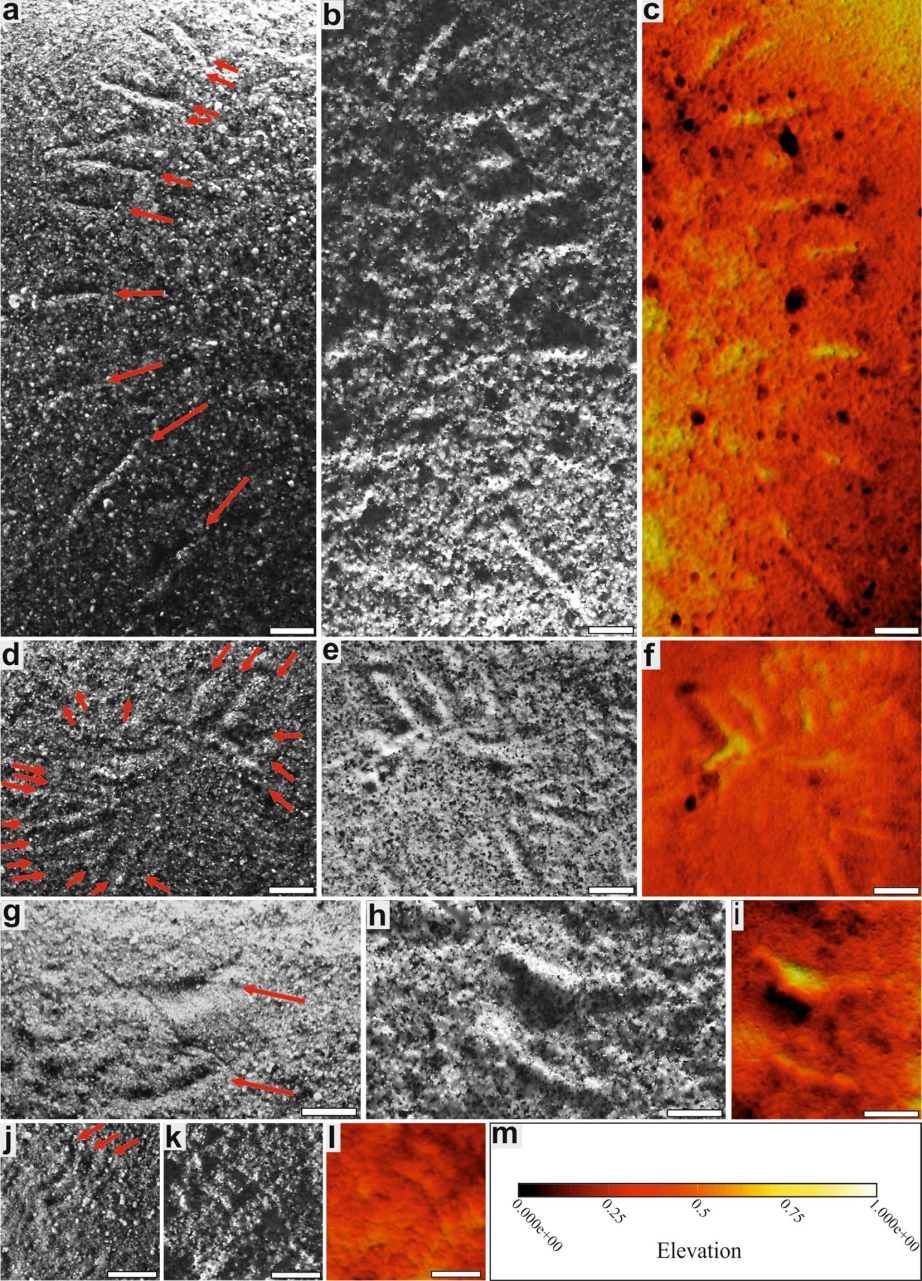
Traces produced by autotomized arms of *Metacrinus rotundus*. (**a**–**c**) Straight deep grooves arranged in radiating group; (**d**–**f**) a few sets of straight parallel grooves and furrows inclined at different angles to each other; (**g**–**i**) two large arched grooves; (**j**–**l**) small short parallel furrows. (**a**, **d**, **g**, **j**) Photographs of sediment surface; (**b**, **e**, **h**, **k)** photographs of gypsum casts; (**c**, **f**, **i**, **l**) false-color depth maps of gypsum casts (for source data see the Open Science Framework, https://osf.io/b8zu2/project ”3D models of crinoid traces”, files SOM7_ichno_3D, SOM8_ichno_3D, SOM9_ichno_3D); (**m**) color scale of elevation. Large and small arrows indicate deep grooves left by the arm and shallow furrows made by pulling the pinnules along the substrate, respectively. Scale bars = 1 cm.

Following autotomy of the arm, within a few seconds, its activity increased, i.e., it started to thrash mostly in the oral–aboral plane. Both frequency and style of flexions (sluggish writhing and violent flicks; electronic supplementary movies S1–S4) and the duration of movement varied (~ several hours to up to about 7 days). However, only in the first hours after autotomy, detached arms showed the highest activity. The frequency and amplitude of motions decreased with time; i.e., after several hours autotomized arms remained in place; their activity could still be recorded but they did not produce any traces. In the first hours after autotomy, each autotomized arm produced two major types of traces on the sediment surface, which can be closely associated with each other (Fig. 1):

1. straight to slightly arched grooves [1.5–3 cm long (mean: 2.1 cm); 0.2–0.6 cm wide (mean: 0.3 cm); ~ 0.1 cm deep) usually arranged in radiating groups (Fig. 1a–c, g–i); the grooves may be inclined at different angles to each other (the angles between adjacent grooves ranging from 9° to 28°) and are separated from each other by a distance of 0.9–2.8 cm (measured in the widest distance between two ends of neighbouring grooves). The length-to-width ratio of these grooves ranges from 3.8 to 10.9 (mean: 6.8). These traces were left by a rotating and more or less rhythmic movement of the flicking arm that placed the most pressure on the substrate with its median-distal arm part.
2. sets of thin and parallel (locally curving) furrows [0.3–1.7 cm long (mean: 1.1 cm), 0.04–0.17 cm wide (mean: 0.1 cm), ~ 0.03 cm deep) (Fig. 1d–f, j–l)]; these sets may be inclined at different angles to each other, locally forming a herringbone pattern. The length-to-width ratio of these furrows ranges from 4.1 to 33.6 (mean: 11.4). These traces were made by pulling the pinnules along the substrate.

**Figure 2:**
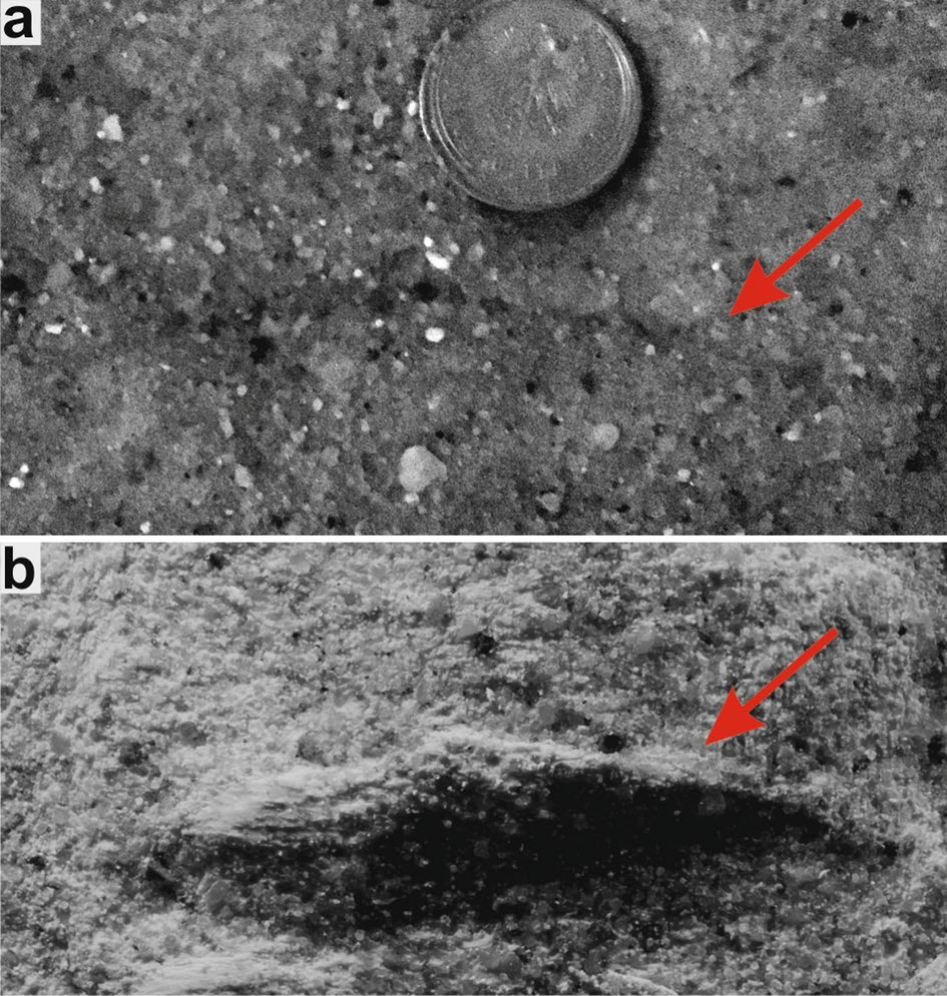
Current-induced sole markings (arrows) left on the sediment surface. (**a**) Photograph of sediment surface; (**b**) photograph of gypsum cast.

Current-produced sole markings, resulting from the more or less continuous contact of the arm with the sediment surface (electronic supplementary movies S5, S6), display a very different morphology (Fig. 2). These grooves are continuous, long (4–6 cm), generally linear, deep (~ 0.2 to 0.4 cm) and run parallel to the flow direction.

## Trace fossils

**Figure 3:**
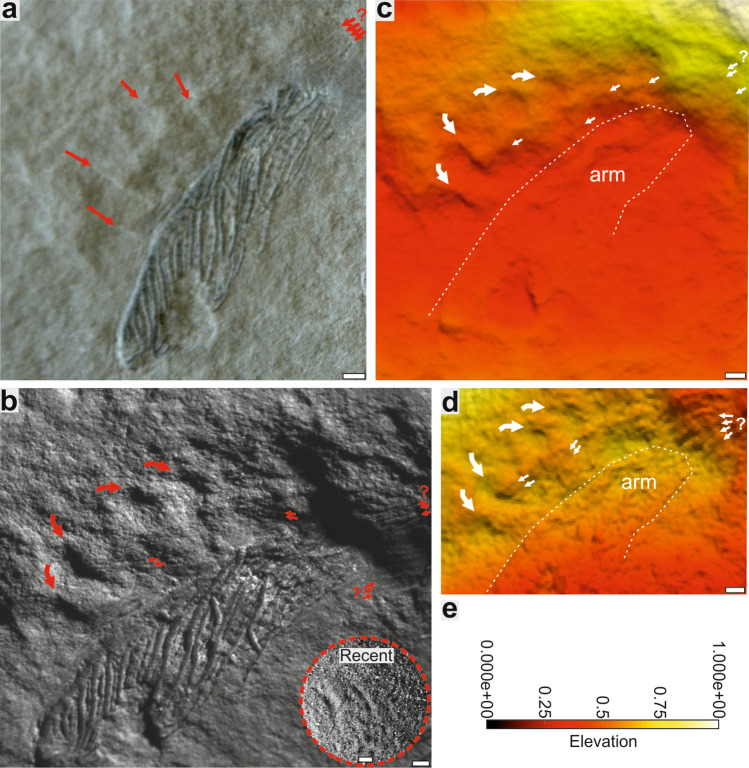
Possible trace fossils associated with articulated piece of arm of *Holocrinus* sp. (UBGD 30564; Thaynes Group, Lower Triassic; collection of Université de Bourgogne, Géologie Dijon, France). (**a**) Slab preserving articulated fragment of arm and associated traces (arrows); a photograph taken using a camera fixed to a tripod. (**b**) Slab preserving articulated fragment of arm and arched grooves (curved red arrows) arranged in radiating group and a few sets of indistinct straight parallel furrows (straight small red arrows with a question mark) and analogical traces (in dotted circle) produced by autotomized arms of Recent isocrinid; a photograph taken using a camera attached to an optical microscope. (**c**) False-color depth map of a slab; (**d**) false-color depth map of a slab acquired from photographs taken from binocular microscope (elevation has been increased (× 2) to enhance depth contrast); (**e**) color scale of elevation. For source 3D data see the Open Science Framework, https://osf.io/b8zu2/project ”3D models of crinoid traces”, files SOM10_old, files SOM11_newest. Scale bars = 1 mm; scale bar in dotted circle = 1 cm.

Close examination of Lower Triassic slabs from the Thaynes Group (Spathian, west of the city of Paris, Idaho, USA^18^) revealed similar traces associated with isolated articulated fragment of arm of *Holocrinus* sp. (Fig. 3). Given their size, morphology and close association with the crinoid arm, it appears that these traces could have been produced by thrashing movements of shed arm. However, the scarcity and imperfect state of preservation make the erection of the new ichnospecies impossible.

### Description

One arm fragment is associated with slightly arched grooves [~ 1.9 to 3.2 mm long (mean: 2.3), ~ 0.5 to 0.8 mm wide (mean: 0.55)] arranged in a radiating group (the angles between adjacent grooves range from 12° to 24°). These grooves are separated from each other by a distance of 1.9–2.6 mm (measured in the widest distance between two ends of neighbouring grooves). Dimensionally, the mean length-to-width ratio of these grooves (4.3) falls well within the range of this ratio for the grooves produced by the median-distal arm part during our neoichnological experiments. These grooves are associated with a few sets of indistinct thin and parallel (locally slightly curving) furrows [1.8–3.2 mm long (mean: 2.4), 0.07–0.13 mm wide (mean: 0.1)] of uncertain origin. Notably, however, the mean length-to-width ratio of these furrows (25.5) falls within the range of this ratio reported in our neoichnological experiments.

### Remarks

As no ethological term exists for the trace fossils produced by post-autotomy thrashing movements of shed appendages we herein propose a new ethological category, autotomichnia (from the Greek auto- "self-" and tome "severing", αὐτοτομία). Although thrashing behaviour occurs in a wide range of vertebrate and invertebrate taxa^1,2,19–22^, autotomichnia are probably rare in the fossil record, and its identification is difficult if the traces are not found in association with the “producer”. Such cases, however, are not likely to be common given that shed appendages displaying movements attract attention of the predators which attempt to consume them. The only ichnospecies ascribed to activity of crinoids described so far is *Krinodromos bentou*^23^. This ichnotaxon, however, in contrast to the presently described traces, refers to unusual locomotion trail, and shows different morphology in the form of two bordering grooves combined with pushed sediment piles, and a central flat area or a narrow winding furrow. Possible explanation for the fossil traces reported herein is that they simply represent sole markings produced by a dead arm moved along by a current. However, these Triassic traces bear no morphological resemblance to the experimentally induced abiotic sedimentary structures (Fig. 1 vs Fig. 2).

## Discussion

It has been argued that ichnology can provide valuable insights on the evolutionary origins of behaviors and on many of their functional and adaptive aspects^24–26^. Our experiments showed that autotomized arms of stalked crinoids (isocrinids) display vigorous movements similar to that observed in shed arms of brittlestars^21^. They produce distinct traces on the sediment surface, which may have some potential to be preserved as trace fossils. This finding opened new perspectives for tracing the origin of thrashing behaviour in crinoids. Indeed, our examination of fossil material from the Thaynes Group suggests that similar traces are associated with isolated articulated fragment of arm of the oldest (~ 250.6 Ma) stem-group isocrinids (*Holocrinus*). The fact that this arm fragment lacks distal arm tip and some distal pinnules is suggestive that it was autotomized due to predatory attack prior to rapid burial rather than another type of disturbance (e.g., storm). It must be emphasized that although abiotic factors (such as high current velocities induced by storms or other environmental trauma) may lead to arm loss, most autotomy in natural settings results from biotic interactions^27^. This is consistent with recent data^28^ which showed that the thruster-produced extreme flow or suction force did not lead to arm loss or any other type of injury in deep-water, stalked crinoid *Democrinus* sp.

Trace fossils described herein suggest that the origin of thrashing behaviour in crinoids could be traced back to at least the Early Triassic. The question of whether this behaviour could have appeared in the Paleozoic crinoids (independently in several clades or in one lineage and was then inherited by the post-Paleozoic descendants) is presently unclear. However, it should be emphasized that, in contrast to the highly flexible muscular arms of recent and post-Paleozoic crinoids in which syzygial or cryptosyzygial ligamentary articulations specifically designed for autotomy are localized, many Paleozoic crinoids possessed primitive arms with limited flexibility^6^. Although regeneration is frequently documented in Paleozoic crinoids^29^, most of them, if not all, have not yet developed autotomy planes^6,11,30^. This suggests that they lacked autotomy abilities, or at least that they were less specialized in that regard^30^.

The end-Permian mass extinction profoundly influenced the evolutionary history of crinoids, not only through the demise of major Paleozoic crinoid groups but also through changes in their functional morphology^31,32^. Notably, post-Paleozoic crinoids are considered to have descended from only a single survivor (i.e., a cladid ancestor—ampelocrinids)^33,34^. In the Middle Triassic, however, they rebounded and underwent a major radiation resulting in the appearance of several motile taxa showing many anti-predatory morphological and behavioral innovations to increased predation pressure during the Mesozoic Marine Revolution^35–39^. Holocrinids are among the first crinoids to appear in the aftermath of the end-Permian mass extinction^40^. These crinoids display many adaptations to benthic and nektonic predators. For instance, they developed specialized rupture points at the distal nodal facets in their stalk, allowing them to free themselves of the sea bottom, crawl with the aid of highly flexible muscular arms and re-attach^41–43^. Furthermore, they elaborated localized autotomy planes (cryptosyzygies) in the arms^38,44^, which in Recent isocrinid descendants greatly reduce mortality and arm damage^16^. Herein it is suggested that holocrinids might have also displayed post-autotomy arm thrashing. Interestingly, in some lizards, it has been observed that their wildly thrashing autotomized tails (in contrast to taxa having a much lower rate of tail thrashing) consistently distract attention of predator away from the escaping lizard^45,46^ and increase predator-handling time, providing additional opportunity to escape^47,48^. Intriguingly, however, in some lacertid lizards, duration of thrashing between different species, shows little variation, either because it is less costly, or because the underlying physiological pathways are evolutionary conservative^49^. Notwithstanding, the high numbers of shed lizard tails are commonly found in predator stomachs^50,51^. All these indicate that caudal autotomy and thrashing behaviour in these vertebrates is an effective antipredatory strategy. Accordingly, Wilkie^21^ argued that thrashing behaviour in ophiuroids is also likely to be an effective decoy against fish predators. Consequently, we hypothesize that this behaviour in crinoids also probably acts as a defense strategy to distract predator attention, and/or to increase predator handling time, providing additional getaway chances to crinoids. A moving autotomized arm might itself be sufficient attraction as a food source. Accordingly, acquisition of thrashing behaviour in the Early Triassic crinoids may underscore the magnitude of the anti-predatory traits that occurred during the Mesozoic Marine Revolution, which had already started soon after the end-Permian extinction^52,53^. The thrashing behaviour along with the specific arm construction consisting of two types of articulations (muscular ones playing a great role in locomotion and postural changes by maximizing arm flexibility, and strictly ligamentary (crypto-)syzygial ones, specifically designed for autotomy) have been maintained in isocrinid/comatulid descendants up to the present.

Given the scarcity and imperfect state of preservation of the trace fossils described herein, further ichnological findings are needed to fully test the above hypotheses. Results of neoichnological experiment presented herein highlight the preservation potential of thrashing behaviour of crinoid arms, thus rock slabs preserving detached crinoid arms are worthy of in-depth investigation.

## Methods

Movements of the autotomized arms of crinoids were observed using a self-made underwater camera that was placed into a small transparent container with a power bank. A camera was constructed on the basis of small single-board computer—Raspberry Pi 3 device with connected V2 Camera Module and combined with the source of red LED light. Both continuous (~ 30 to 35 min) and time-lapse movies (~ 10 to 11 h; at the frequency: one photograph taken per 1 min) were captured (electronic supplementary movies S1–S4). The traces on the sediment were photographed under different light conditions, and then gypsum casts were made. 3D models were acquired with Shining 3D EinScan Pro 2X 3D scanner fixed on a tripod, EXScan Pro 3.2.0.2 software, and then processed with Meshlab 1.3.3, Blender 2.82 and ParaView 5.80 to obtain the false-color depth maps.

Two sets of photographs of the trace fossils associated with of the holocrinid arm fragment were taken under different light conditions [by mean of a camera fixed to a tripod (Fig. 3a) and with a camera attached to an optical microscope (Fig. 3b)]. Two 3D models and false-color depth maps for each set were acquired by means of photogrammetric technique using Visual SFM 0.5.26 and the MeshLab 1.3.3 or Agisoft Photoscan 1.2.0.

### Ethics

All experiments on live stalked crinoids were performed and approved by the University of Nagoya, where no special ethics approval is required for the maintenance and handling of this particular species. Nevertheless, our research conformed to the ethical principles of replacement, reduction, refinement and minimization of animal suffering following the guidelines reported in the European Directive 2010/63/EU.

## Supplementary information

Supplementary Video 1.

Supplementary Video 2.

Supplementary Video 3.

Supplementary Video 4.

Supplementary Video 5.

Supplementary Video 6.

Supplementary Information.

## Data Availability

The source files (3D models) of Recent and fossil traces are openly available in the Open Science Framework, project „3D models of crinoid traces”, at https://osf.io/b8zu2/.

## References

[1] Maginnis TL (2006). The cost of autotomy and regeneration in animals: a review and framework for future research. Behav. Ecol..

[2] Arnold EN (2007). Evolutionary aspects of tail shedding in lizards and their relatives. J. Nat. Hist..

[3] Oji T (1996). Is predation intensity reduced with increasing depth? Evidence from the west Atlantic stalked crinoids *Endoxocrinus parrae* (Gervais) and implications for the Mesozoic marine revolution. Paleobiology.

[4] Donovan SK, Pawson DL (1997). Proximal growth of the column in bathycrinid crinoids (Echinodermata) following decapitation. Bull. Mar. Sci..

[5] Wilkie IC (2001). Autotomy as a prelude to regeneration in echinoderms. Microsc. Res. Tech..

[6] Baumiller TK (2008). Crinoid ecological morphology. Annu. Rev. Earth Planet. Sci..

[7] Wilkie IC, Barbaglio A, Maclaren WM, Candia Carnevali MD (2010). Physiological and immunocytochemical evidence that glutamatergic neurotransmission is involved in the activation of arm autotomy in the featherstar *Antedon mediterranea* (Echinodermata: Crinoidea). J. Exp. Biol..

[8] Meyer DL, Burke DR, Mladenov DP, Lambert P, Parsley LR (1988). Crinoids as renewable resources: rapid regeneration of the visceral mass in a tropical reef-dwelling crinoid from Australia. Echinoderm biology.

[9] Shibata TF, Oji T (2005). Autotomy and arm number increase in *Oxycomanthus japonicus* (Echinodermata, Crinoidea). Invertebr. Biol..

[10] Mozzi D, Dolmatov IY, Bonasoro F, Candia Carnevali MD (2006). Visceral regeneration in the crinoid *Antedon mediterranea*: basic mechanisms, tissues and cells involved in gutre growth. Cent. Eur. J. Biol..

[11] Gahn FJ, Baumiller TK (2005). Arm regeneration in Mississippian crinoids: evidence of intense predation pressure in the Paleozoic?. Paleobiology.

[12] Kitazawa K, Oji T (2010). Particle selection by the sea lily *Metacrinus rotundus* Carpenter 1884 (Echinodermata, Crinoidea). J. Exp. Mar. Biol. Ecol..

[13] Kitazawa K, Oji T (2014). Active feeding behavior of and current modification by the sealily *Metacrinus rotundus* Carpenter 1884 (Echinodermata: Crinoidea). J. Exp. Mar. Biol. Ecol..

[14] Brom KR, Oguri K, Oji T, Salamon MA, Gorzelak P (2018). Experimental neoichnology of crawling stalked crinoids. Swiss J. Palaeontol..

[15] Holland ND, Grimmer JC (1981). Fine structure of syzygia articulations before and after arm autotomy in *Florometra serratissima* (Echinodermata: Crinoidea). Zoomorphology.

[16] Oji T, Okamoto T (1994). Arm autotomy and arm branching pattern as anti-predatory adaptations in stalked and stalkless crinoids. Paleobiology.

[17] Saucède T, Vennin F, Fara E, Olivier N, The Paris Biota Team (2019). A new holocrinid (Articulata) from the Paris Biota (Bear Lake County, Idaho, USA) highlights the high diversity of Early Triassic crinoids. Geobios.

[18] Brayard A, Krumenacker LJ, Botting JP, Jenks JF, Bylund KG, Fara E (2017). Unexpected Early Triassic marine ecosystem and the rise of the Modern evolutionary fauna. Sci. Adv..

[19] Bassler U (1984). A movement generated in the peripheral nervous system: rhythmic flexion by autotomized legs of the stick insect *Cunicuuna impigra*. J. Exp. Biol..

[20] Dial BE, Fitzpatrick LC (1981). The energetic costs of tail autotomy to reproduction in the lizard *Coleonyx brevis* (Sauria: Gekkonidae). Oecologia.

[21] Wilkie C (1978). Arm autotomy in brittlestars (Echinodermata : Ophiuroidea). J. Zool. Lond..

[22] Norman MD, Finn J (2001). Revision of the *Octopus horridus* species-group, including erection of a new subgenus and description of two member species from the Great Barrier Reef, Australia. Invertebr. Tax..

[23] Neto de Carvalho C, Pereira B, Klompmaker A, Baucon A, Moita JA, Pereira P (2016). Running crabs, walking crinoids, grazing gastropods: behavioral diversity and evolutionary implications of the Cabeço da Ladeira Lagerstätte (Middle Jurassic, Portugal). Comun. Geol..

[24] Dzik J (2005). Behavioral and anatomical unity of the earliest burrowing animals and the cause of the ‘Cambrian explosion’. Paleobiology.

[25] Niedźwiedzki G, Szrek P, Narkiewicz K, Narkiewicz M, Ahlberg PE (2010). Tetrapod trackways from the early Middle Devonian of Poland. Nature.

[26] Plotnick RE (2012). Behavioral biology of trace fossils. Paleobiology.

[27] Gahn FJ, Baumiller TK (2010). Evolutionary History of regeneration in crinoids (Echinodermata). Integr. Comp. Biol..

[28] Veitch MA, Baumiller TK (2012). Low predation intensity on the stalked crinoid *Democrinus* sp. (Echinodermata) in Roatán, Honduras reveals deep water as likely predation refuge. Bull. Mar. Sci..

[29] Baumiller TK, Gahn FJ (2004). Testing predation-driven evolution using Mid-Paleozoic crinoid arm regeneration. Science.

[30] Oji T (2001). Fossil record of echinoderm regeneration with special regard to crinoids. Microsc. Res. Tech..

[31] Foote M (1995). Morphological diversification of Paleozoic crinoids. Paleobiology.

[32] Twitchett RJ, Oji T (2005). Early Triassic recovery of echinoderms. C. R. Palevol.

[33] Simms MJ, Hess H, Ausich WI, Brett EC, Simms JM (1999). Systematics, phylogeny and evolutionary history. Fossil crinoids.

[34] Simms MJ, Sevastopulo GD (1993). The origin of articulate crinoids. Palaeontology.

[35] Baumiller TK, Messing CG (2007). Stalked crinoid locomotion and its ecological and evolutionary implications. Palaeontol. Electron..

[36] Baumiller TK, Mooi R, Messing CG (2008). Urchins in a meadow: paleobiologial and evolutionaryimplications of cidaroidpredation on crinoids. Paleobiology.

[37] Baumiller TK, Salamon MA, Gorzelak P, Mooi R, Messing ChG, Gahn FJ (2010). Post-Paleozoic crinoid radiation in response to benthic predation preceded the Mesozoic marine revolution. Proc. Natl Acad. Sci. USA.

[38] Hagdorn H (2011). Triassic: the crucial period of post Palaeozoic crinoid diversification. Swiss J. Palaeontol..

[39] Salamon MA, Niedźwiedzki R, Gorzelak P, Lach R, Surmik D (2012). Bromalites from the Middle Triassic of Poland and the rise of the Mesozoic marine revolution. Palaeogeogr. Palaeoclimatol. Palaeoecol..

[40] Salamon MA, Gorzelak P, Hanken NM, Riise HE, Ferré B (2015). Crinoids from Svalbard in the aftermath of the end−Permian mass extinction. Pol. Polar Res..

[41] Baumiller TK, Hagdorn H (1995). Taphonomy as a guide to functional morphology of Holocrinus, the first post-Paleozoic crinoid. Lethaia.

[42] Gorzelak P (2018). Microstructural evidence for stalk autotomy in Holocrinus—the oldest stem-group isocrinid. Palaeogeogr. Palaeoclimatol. Palaeoecol..

[43] Gorzelak, P. & Salamon, M. A. Holocrinus—The oldest stem-group isocrinid with stalk shedding and crawling abilities: evidence from taphonomy, microstructure and trace fossils*.* In *11th North American Paleontological Conference Program with Abstracts* (eds Droser, M., Hughes, N., Bonuso, N., Bottjer, D., Eernisse, D., Gaines, R., Hendy, A., et al.) 153–154 (2019).

[44] Rasmussen, H. W. *Articulata* (eds Moore, C. R. & Teichert, C.) T813–T928 (Boulder and Lawrence, 1978).

[45] Vitt LJ, Congdom JD, Dickson NA (1977). Adaptive strategies and energetics of tail autotomy in lizards. Ecology.

[46] Daniels CB, Flaherty SP, Simbotwe MP (1986). Tail size and effectiveness of autotomy in a lizard. J. Herpetol..

[47] Dial BE, Fitzpatrick LC (1983). Lizard tail autotomy: Function and energetics of postautotomy tail movement in *Scincella lateralis*. Science.

[48] Medel RG, Jiminez JE, Fox SF, Jaksic FM (1988). Experimental evidence that high-population frequencies of lizard tail autotomy indicates inefficient predation. Oikos.

[49] Pafilis P, Foufopoulos J, Poulakakis N, Lymberakis P, Valakos ED (2009). Tail shedding in island lizards [Lacertidae, Reptilia]: decline of antipredator defenses in relaxed predation environments. Evolution.

[50] Pianka ER (1969). Notes on the biology of *Varanus caudolineatus* and *Varanus gilleni*. West. Aust. Nat..

[51] Arnold EN, Gans C, Huey RB (1988). Caudal autotomy as a defense. Biology of the Reptilia.

[52] Scheyer TM, Romano C, Jenks J, Bucher H (2014). Early Triassic marine biotic recovery: the predators perspective. PLoS ONE.

[53] Brachaniec T, Niedźwiedzki R, Surmik D, Krzykawski T, Szopa K, Gorzelak P, Salamon MA (2015). Coprolites of marine vertebrate predators from the Lower Triassic of southern Poland. Palaeogeogr. Palaeoclimatol. Palaeoecol..

