# Supplementary material for: Experimental neoichnology of post-autotomy arm movements of sea lilies and possible evidence of thrashing behaviour in Triassic holocrinids
Source: Sci Rep. 2020 Sep 15;10:15147. doi: 10.1038/s41598-020-72116-1 (PMC7492279; doi:10.1038/s41598-020-72116-1)
Supplement: Supplementary file 7 — Supplementary Information. [file 41598_2020_72116_MOESM7_ESM.docx]

**Supplementary information for**

**Experimental neoichnology of post-autotomy arm movements of sea lilies and possible evidence of thrashing behaviour in Triassic holocrinids**

**Przemysław Gorzelak^1*^, Mariusz A. Salamon^2^, Krzysztof Brom^2^, Tatsuo Oji^3^, Kazumasa Oguri^4^, Dorota Kołbuk^1^, Marek Dec^5^, Tomasz Brachaniec^2^, Thomas Saucède^6^**

*^1^Institute of Paleobiology, Polish Academy of Sciences, Warsaw, Poland,* [*pgorzelak@twarda.pan.pl*](about:blank) *(corresponding author)*

*^2^Faculty of Natural Sciences, University of Silesia in Katowice, Sosnowiec, Poland*

*^3^University Museum, Nagoya University, Furo-cho, Nagoya 464-8601, Japan*

*^4^Japan Agency for Marine-Earth Science and Technology (JAMSTEC), 2-15 Natsushima-cho, Yokosuka 237-0061, Japan*

*^5^Polish Geological Institute – National Research Institute, Warsaw, Poland*

^6^Biogéosciences. UMR CNRS 6282*, Université Bourgogne Franche-Comté, Dijon, France*

**Supplementary Movies**

Supplementary Movie 1. Post-autotomy arm movements of sea lily *Metacrinus rotundus*.

Supplementary Movie 2. Post-autotomy arm movements of sea lily *Metacrinus rotundus*.

Supplementary Movie 3. Post-autotomy arm movements of sea lily *Metacrinus rotundus* (a time-lapse movie; one photograph taken per one minute).

Supplementary Movie 4. Post-autotomy arm movements of sea lily *Metacrinus rotundus* (a time-lapse movie; one photograph taken per one minute).

Supplementary Movie 5. Current-induced transport of isolated dead arm of sea lily *Metacrinus rotundus* being dragged with the pinnules facing downstream.

Supplementary Movie 6. Current-induced Transport of isolated dead arm of sea lily *Metacrinus rotundus* being dragged with the pinnules facing upstream.
